# H_2_O_2_-dependent substrate oxidation by an engineered diiron site in a bacterial hemerythrin†
†Electronic supplementary information (ESI) available: Information on materials, instrumentation, experimental details and additional data on preparation of proteins, crystal structure analysis, resonance Raman and FTIR spectroscopy, reaction of reduced I119H with O_2_, consumption of H_2_O_2_, and oxidation reactions of guaiacol and 1,4-cyclohexadiene. The atomic coordinates and structure factors (PDB code 3WHN) have been deposited into the Protein Data Bank, http://www.rcsb.org/. See DOI: 10.1039/c3cc48108e



**DOI:** 10.1039/c3cc48108e

**Published:** 2014-01-08

**Authors:** Yasunori Okamoto, Akira Onoda, Hiroshi Sugimoto, Yu Takano, Shun Hirota, Donald M. Kurtz, Yoshitsugu Shiro, Takashi Hayashi

**Affiliations:** a Department of Applied Chemistry , Graduate School of Engineering , Osaka University , Suita , Osaka 565-0871 , Japan . Email: onoda@chem.eng.osaka-u.ac.jp ; Email: thayashi@chem.eng.osaka-u.ac.jp; b Biometal Science Laboratory , RIKEN SPring-8 Center , Sayo , Hyogo 679-5148 , Japan; c Institute for Protein Research , Osaka University , Suita , Osaka 565-0871 , Japan; d Graduate School of Materials Science , Nara Institute of Science and Technology , Ikoma 630-0192 , Japan; e Department of Chemistry , University of Texas at San Antonio , San Antonio , Texas 78249 , USA

## Abstract

The O_2_-binding carboxylate-bridged diiron site in DcrH-Hr was engineered in an effort to perform the H_2_O_2_-dependent oxidation of external substrates. A His residue was introduced near the diiron site in place of a conserved residue, Ile119. The I119H variant promotes the oxidation of guaiacol and 1,4-cyclohexadiene upon addition of H_2_O_2_.

## Abstract 2

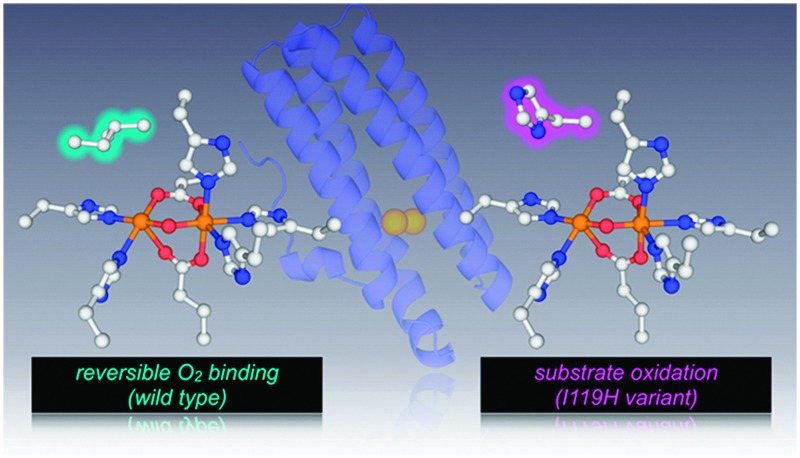
The O_2_-binding carboxylate-bridged diiron site in DcrH-Hr with an engineered His residue in place of Ile119 promotes the oxidation of guaiacol and 1,4-cyclohexadiene upon addition of H_2_O_2_.

O_2_ is utilized in several non-heme diiron-carboxylate proteins performing essential biological processes ranging from transport of O_2_ (hemerythrin, Hr),^1,2^ hydroxylation of C–H bonds (methane monooxygenase hydroxylase),^3^ generation of a tyrosyl radical for DNA synthesis (ribonucleotide reductase),^4^ and reduction of unsaturated fatty acids (Δ9 desaturase).^5^ Despite these functional differences, all of these proteins have a carboxylate-bridged diiron active site within a four-helix bundle protein folding motif.^2a,6^ The coordination sphere and the environment surrounding the diiron site in these proteins have apparently evolved to optimize a variety of reactions with O_2_.

Engineering of the first or the second coordination sphere of the diiron site^7^ constitutes one approach to understanding the effects of the protein environment, which distinguish reversible O_2_ binding from O_2_ activation. As a target protein for engineering the coordination sphere, we have selected DcrH-Hr,^8^ which contains the histidine-rich coordination sphere and exhibits spectroscopic properties characteristic of all Hrs, but contains a larger substrate-accessible tunnel within the four-helix bundle compared to classical Hrs.^9,10^ As for classical Hrs, the diiron site of DcrH-Hr reversibly binds O_2_ but shows more rapid autoxidation. Recently, we succeeded in altering the first coordination sphere of the diiron site in DcrH-Hr by replacing residue Ile119 located close to the exogenous ligand binding site with a Glu residue.^11^ The I119E variant showed altered properties of exogenous ligand binding and redox behavior in the wild type DcrH-Hr (WT). Herein, we report another engineered variant DcrH-Hr, I119H, of the same residue, in which alteration of the coordination sphere generates the capability of H_2_O_2_-dependent oxidation of exogenous substrates.

I119H was expressed from *Escherichia coli* and purified as a holo-form containing approximately 1.6 iron atoms per protein, as determined by ferrozine analysis.^12^ The diferric form (met-I119H) exhibited two absorption maxima at 325 and 375 nm, which are well-established to arise from oxo to ferric LMCT transitions in the oxo–dicarboxylate-bridged met-Hrs.^1b,c,8^ Resonance Raman (rR) spectra of I119H gave a characteristic peak at 499 cm^–1^ for the symmetric stretching Fe–O_oxo_–Fe vibrational mode.^8^ The met-I119H protein was crystallized, and the structure of the oxo-bridged diiron site^13^ was determined by X-ray crystallographic analysis at a resolution of 1.9 Å. The overall structure of the met-I119H is similar to that of the met form of WT (met-WT) (RSMD value, 0.324).^10^ Surprisingly, H119 coordinates to Fe1 in the crystal structure in place of the conserved H118 ligand residue in WT (Fig. 1). The coordination geometry of other His residues and carboxylate ligands is almost identical to that of met-WT (Fig. S1, ESI†). A computational study of the I119H diiron site yielded alternative structures in which either H118 or H119 coordinates to the Fe1 (Fig. 2).^14^ It is, thus, expected that either H118 or H119 coordination would be stable in I119H in solution.

**Fig. 1:**
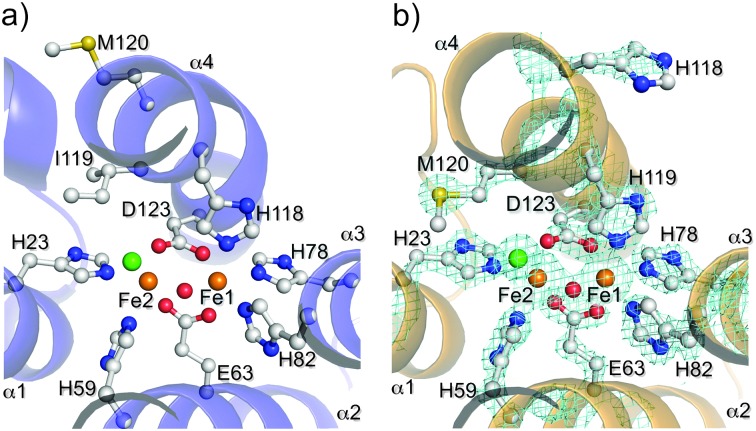
Crystal structures of (a) met-WT (PDB code: 3AGT) and (b) met-I119H (iron in brown, oxygen in red, nitrogen in blue, carbon in white, sulfur in yellow, and chloride in green). The 2*F*_obs_ – *F*_calc_ electron density in light blue grid (contoured at 1.5*σ*) around the diiron site. The structure drawings were generated in PyMOL.^15^

**Fig. 2:**
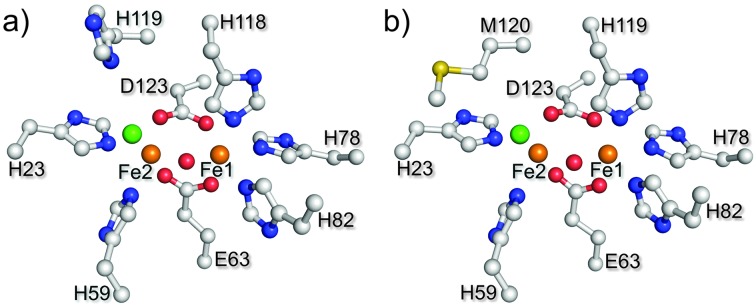
Depiction of computationally optimized structures of I119H diiron site models (a) where H118 coordinates to Fe1 and (b) where H119 coordinates to Fe1 (iron in brown, carbon in white, oxygen in red, nitrogen in blue, chloride in green, and sulfur in yellow).

The exogenous ligand binding properties of the met-I119H diiron site were investigated using azide, which is known to coordinate to Fe2 in place of chloride in met-WT.^9^ The azide adduct of met-WT exhibits a UV-vis absorption at 443 nm,^8^ while that of I119H showed a shifted absorption maximum at 425 nm, indicating a change in conformation or orientation of the bound azide ligand (Fig. S2, ESI†). The rR spectrum of I119H showed the Fe–N_3_ stretching vibration at 361 cm^–1^, which was confirmed by ^14^N_3_^–^/^15^N_3_^–^ isotope labeling experiments (Fig. 3a). The Fe–N_3_ stretching vibration of I119H is downshifted compared to that of the WT at 372 cm^–1^.^8^ This finding suggests weaker Fe–N_3_ bonding in I119H than that observed in WT.

**Fig. 3:**
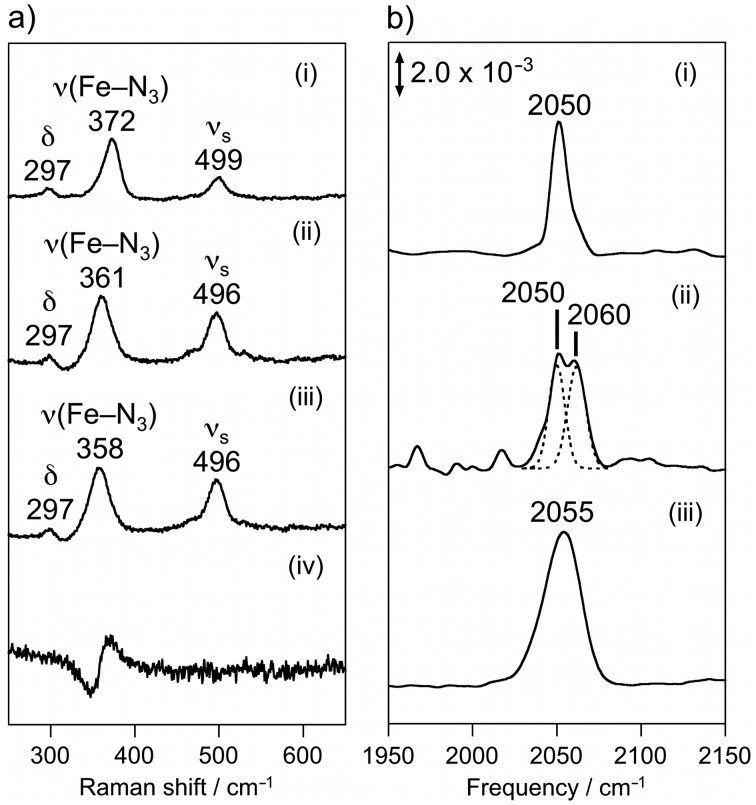
(a) Resonance Raman spectra of the azide adduct of (i) met-WT with Na^14^N_3_, (ii) met-I119H with Na^14^N_3_, (iii) met-I119H with Na^15^N_3_, and (iv) the difference spectrum ^15^N_3_ minus ^14^N_3_ adducts of met-I119H. Peaks are labeled according to their vibrational assignments, as described in the text. (b) FTIR spectra of the azide adduct of (i) met-WT at 5 K, (ii) met-I119H at 5 K, and (iii) met-I119H at 25 °C.

Additional structural details of the bound azide ligand were obtained from FTIR spectroscopy in the region of the asymmetric stretch of the coordinated azide *ν*_as_(NNN). The azide adduct of met-WT showed a *ν*_as_(NNN) band at 2050 cm^–1^ (Fig. 3b). The isotope labeling experiment using ^15^NN_2_ indicates that the terminal nitrogen of the azide ligand binds in a η^1^ fashion, as expected from the X-ray crystal structure of the met-WT azide adduct.^1,9,16^ The azide adduct of met-I119H exhibits two *ν*_as_(NNN) bands at 2050 and 2060 cm^–1^ at 5 K (Fig. 3b). The newly observed band, 10 cm^–1^-higher than that of met-WT, suggests a resonance form shifted farther from the symmetric [Fe^3+^–N^–^ =N^+^ =N^–^] towards the asymmetric [Fe^3+^–N^2–^–N^+^ ≡N]. The increased asymmetry of the two N–N bonds could be stabilized by a hydrogen bond interaction with the 1N atom of the coordinated azide, possibly with the displaced His119 ligand.^17^ No such hydrogen bonding interaction is observed in the structure of the met-WT azide adduct.^9^ The two *ν*_as_(NNN) bands observed at 5 K coalesced at 25 °C (Fig. 3b). Based on this observation together with the crystal structure and calculated optimized structures, we propose two conformations for I119H: H118off/H119on and H118on/H119off. In the latter case, the H119 residue is capable of forming a hydrogen bonding interaction with an exogenous ligand.

We evaluated the reactivity of I119H toward H_2_O_2_ as a potential exogenous ligand, which, like azide, could form a hydrogen bond with the pendant H119 residue. The met-WT and met-I119H in 50 mM HEPES (pH 7.0) were individually treated with 50 eq. of H_2_O_2_ from an aqueous solution, and the amount of H_2_O_2_ consumed was determined by the iodometry method. Disproportionation of H_2_O_2_ into O_2_ and H_2_O has been reported to occur upon reaction of H_2_O_2_ with a synthetic Hr model complex.^18^ We found that the met-WT reacted with H_2_O_2_ within 10 min with a concomitant evolution of O_2_, which was confirmed by gas chromatography (Fig. S4, ESI†). In addition, the deoxy-WT was oxidized to the met form by H_2_O_2_ (Fig. S5, ESI†). These results indicate that the disproportionation reaction of H_2_O_2_ could proceed *via* a mechanism analogous to that proposed for the Hr model complex (Scheme S1, ESI†). The consumption rate of H_2_O_2_ by I119H was significantly slower than that of WT (Fig. 4a).

**Fig. 4:**
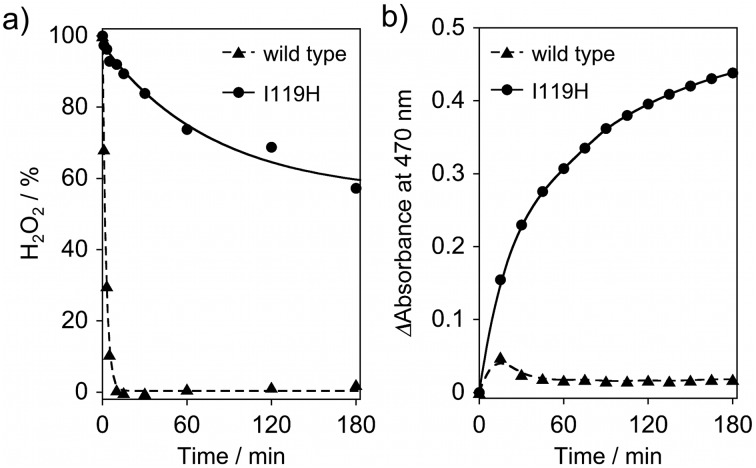
(a) Consumption of H_2_O_2_ in the presence of met-I119H (solid line) and met-WT (dashed line); [protein] = 100 μM, [H_2_O_2_] = 5 mM. All experiments were carried out in 50 mM HEPES (pH 7.0) at 25 °C. (b) Oxidation reaction of guaiacol by met-I119H (solid line) and met-WT (dashed line) at 25 °C. The absorption of the guaiacol product at 470 nm was monitored; [protein] = 100 μM, [guaiacol] = 5 mM, [H_2_O_2_] = 5 mM.

Motivated by the differing reactivity of WT and I119H toward H_2_O_2_, we tested the promotion of oxidation of external substrates by these proteins. Fig. 4b demonstrates that I119H promotes oxidation of guaiacol by H_2_O_2_, as shown by the clear increase in absorption at 470 nm assigned to the oxidized guaiacol product, whereas, under the same conditions, WT does not. Similarly, I119H promotes the oxidation of 1,4-cyclohexadiene by H_2_O_2_, producing benzene as a product determined by GC-MS analysis, whereas WT showed no such reactivity (Fig. S6, ESI†). These findings support the generation of an oxidizing intermediate from the altered diiron site in I119H which cannot be generated in WT. According to the reaction analysis, the oxidized product yields for both the guaiacol and cyclohexadiene reactions are approximately 0.26 mol product per mol of a diiron site under the conditions as described in Fig. 4b. During the substrate oxidation reaction, H_2_O_2_ is also expected to be consumed by its concurrent disproportionation. We confirmed that the oxidation of guaiacol could be continued upon the addition of H_2_O_2_ to the initial reaction solution (Fig. S7, ESI†). The key for the generation of the oxidizing intermediate could be a hydrogen bonding interaction between a hydroperoxide coordinating in a fashion similar to that in oxyHr and the displaced H119 ligand. Previous studies have invoked hydrogen bonding to a hydroperoxide bound to Fe(iii) as promoting formation of a high-valent iron-oxo species.^19^ Support for such a hydrogen bonding interaction comes from the azide ligation experiment monitored by FTIR spectroscopy. An additional contribution could involve dynamic coordination of H118 and H119, which transiently forms a five-coordinated Fe1 site *via* a conformational transition during the reaction. This transient coordination site may allow formation of a μ-1,2-peroxo-diferric species, which is the most commonly proposed precursor to high-valent iron–oxo species in non-heme diiron-carboxylate enzymes.^20^

In conclusion, we have engineered the diiron site of DcrH-Hr to promote the H_2_O_2_-dependent oxidation of exogenous substrates. A His residue introduced proximal to the diiron site will participate in a hydrogen bonding interaction with the exogenous H_2_O_2_ ligand, thereby, activating the peroxide. To the best of our knowledge, this is the first example that successfully converts an O_2_-binding non-heme diiron-carboxylate protein to an oxidatively active one. Efforts to characterize the oxidatively active species of the I119H variant and the further engineering of the diiron site are in progress.

This work was financially supported by Grants-in-Aid for Scientific Research ((C), JSPS KAKENHI Grant Number 205590020, and Innovative Areas “Molecular Activation”, area 2204, MEXT KAKENHI Grant Number 221050130). Y.O. appreciates support from the Research Fellowship of JSPS and Global COE Program “Global Education and Research Center for Bio-environmental Chemistry” of Osaka University. D. M. K., Jr. acknowledges support from the National Institutes of Health (grant R01 GM040388). The computations were performed at the Research Center for Computational Science, Okazaki, Japan.
