# Supplementary material for: H2O2-dependent substrate oxidation by an engineered diiron site in a bacterial hemerythrin
Source: Chem Commun (Camb). 2014 Jan 8;50(26):3421–3. doi: 10.1039/c3cc48108e (PMC3947700; doi:10.1039/c3cc48108e)
Supplement: Supplementary file 1 [file CC-050-C3CC48108E-s001.pdf]

This journal is © The Royal Society of Chemistry 2014

Supporting Information

H_2O_2-dependent Substrate Oxidation by an Engineered Diiron Site in a Bacterial Hemerythrin

Yasunori Okamoto,[a] Akira Onoda,*[a] Hiroshi Sugimoto,[b] Yu Takano,[c] Shun Hirota,[d]

Donald M. Kurtz, Jr.,[e] Yoshitsugu Shiro,[b] and Takashi Hayashi*[a]

[a] Department of Applied Chemistry, Graduate School of Engineering, Osaka University, Suita, Osaka 565-0871, Japan.

[b] Biometal Science Laboratory, RIKEN SPring-8 Center, Sayo, Hyogo 679-5148, Japan.

[c] Institute for Protein Research, Osaka University, Suita, Osaka 565-0871, Japan.

[d] Graduate School of Materials Science, Nara Institute of Science and Technology, Ikoma 630-0192, Japan.

[e] Department of Chemistry, University of Texas at San Antonio, San Antonio, Texas 78249.

This journal is © The Royal Society of Chemistry 2014

Materials and Instruments

Oligonucleotides were obtained from Invitrogen, Inc. Restriction enzymes were obtained from Takara Bio Inc. Nucleotide sequences were determined by FASMAC Co., Ltd. or the University of Texas Health Science Center, San Antonio Nucleic Acids Core Facility. All reagents of the highest guaranteed grade were purchased and used as received unless otherwise noted. Sodium azide-1-^{15}N was purchased from Isotech Laboratories Inc. Distilled water was demineralized by a Barnstead NANOpure DIamond apparatus. Purification of the proteins was performed using a GE Healthcare ÄKTA Purifier system at 4 ºC. The UV−vis experiments were conducted using a Shimadzu UV-3150 double-beam spectrophotometer equipped with a thermostated cell holder with a 0.1 ºC deviation or Shimadzu BioSpec-nano. The FTIR experiments were conducted using a Jasco FT/IR 6100. During FTIR measurements, the sample temperature was maintained at 5 K by an Oxford Instruments continuous liquid helium cryostat equipped with a turbo pump to lower the vapor pressure of the liquid helium. The pH values were monitored with a Horiba F-52 pH meter.

Protein Overexpression and Purification

The expression plasmids of His-tagged and tag-free recombinant DcrH-Hr were prepared by the previously described procedures.[S1] The plasmid containing the gene encoding the I119H variant was constructed by a round-the-horn site-directed mutagenesis protocol using the plasmids for the wild-type as a template. The forward primer overlapping the Ile119 codon, 5'-CAT<u>CAT</u>ATGAAGGAGGACAAGAAGTACGAGGCGTACTTGCGCG-3', where the variant codons are underlined, and reverse primer, 5'-GTTCACCAGCCAGTCGACGAGTCCG-3', were used for the tag-free I119H variant. In a similar way, the forward primer, 5'-GGCTGGTCAACCAC<u>CAC</u>ATGAAAGAAGACAAAAAATATGAAGCG-3' and the reverse primer, 5'-AGTCAACCAGACCGCGCAGCGTGGTC-3', were used for the His-tagged I119H. Each expression plasmid of His-tagged wild-type (WT), I119H, and His-tagged I119H was transformed into *E. coli* BL21(DE3). Twenty-milliliter cultures of the transformed cells in Luria-Bertani broth (LB) containing 50 μg/mL ampicillin were incubated overnight at 37 ºC. The 20-mL cultures were used to inoculate 1-L batches of LB containing ampicillin (50 mg) and 1% (w/v) glucose. The inoculated 1-L cultures were incubated aerobically with vigorous shaking at 37 ºC. When the cultures' OD_{600} reached 1.0, isopropyl β-D-1-thiogalactopyranoside was added to a final concentration of 0.4 mM to induce protein expression. Ammonium iron(II) sulfate (30 mg) was also added to facilitate incorporation of iron into the expressed protein. The cultures were incubated at 25 ºC overnight with shaking, and the cells were harvested by centrifugation.

The harvested cells from 10 L of culture were re-suspended in 200 mL of 50 mM 3-(*N*-morpholino)propanesulfonic acid (MOPS) (pH 7.3) and lysed by sonication. Cell debris was

This journal is © The Royal Society of Chemistry 2014

removed from the lysate by centrifugation at 20000*g* for 60 min, and the yellow supernatant was loaded onto a column packed with 10 mL of TALON metal affinity resin (Clontech Laboratories, Inc.) which was pre-equilibrated in 50 mM MOPS buffer (pH 7.3) containing 250 mM NaCl. After the column was washed with 50 mL of washing buffer (50 mM MOPS (pH 7.3), 10 mM imidazole, 250 mM NaCl), the His-tagged WT or I119H was eluted as a yellow-green band with 50 mL of elution buffer (50 mM MOPS (pH 7.3), 0.5 M imidazole, 0.5 M NaCl). The collected fraction was buffer-exchanged into imidazole-free 50 mM MOPS (pH 8.0) buffer. The iron content in the purified protein was quantified by ferrozine iron analysis.[S2]

For protein crystallography, cells were harvested from 4 L of culture expressing the tag-free I119H. These cells were resuspended in 100 mL of 50 mM MOPS (pH 7.3). The lysate was treated with benzonase nuclease (Novagen) (250U) for 40 min at 25 ºC. The supernatant from centrifugation of the treated lysate was diluted 10-fold with 50 mM MOPS (pH 7.3). The diluted solution was loaded onto an anion-exchange column packed with DEAE Sepharose Fast Flow resin (GE Healthcare), which was pre-equilibrated in 50 mM MOPS (pH 7.3). The flow-through fraction containing I119H was collected and concentrated to 5 mL using an Amicon stirred ultrafiltration cell with a 10-kDa molecular weight cutoff membrane (Millipore). The sample solution was loaded onto a HiTrap Q anion-exchange column, and a flow-through fraction was collected. The sample fraction was loaded onto a Sephacryl S-200 column (GE Healthcare) equilibrated in the same buffer.

The as-purified DcrH-Hr variants were oxidized by addition of at least 10 eq of potassium ferricyanide at 4 ºC for 16 h, and the resulting samples were purified using a HiTrap desalting column (GE Healthcare) equilibrated with 50 mM HEPES (pH 7.0).

Reaction of Reduced I119H with O_2

The met form of I119H (met-I119H) (50 μM) was anaerobically reduced to the deoxy form by the addition of 1 eq of sodium dithionite at 25 ºC. The solution was incubated for 30 min in a glovebox under an N_2 atmosphere. The O_2 gas was bubbled to the resulting solution.

Crystal Structure Analysis

Crystal of met-I119H was grown by hanging drop vapor diffusion by mixing 0.14 μL of a protein solution (38 mg/mL) with 0.14 μL of the reservoir solution (0.1 M Tris-HCl (pH 8.2), 12% (v/v) 2-propanol, 30% (w/v) PEG4000, 0.2 M $CaCl_2$) at 10 ºC. The crystal was then soaked in a cryoprotectant solution (20% ethylene glycol in a reservoir solution) and flash-frozen in liquid nitrogen. Crystal of met-I119H belongs to space group *P*1 with two monomers in asymmetric unit. X-ray diffraction data were collected on the BL41XU beam-line at the SPring-8 synchrotron radiation facility. The data were integrated and scaled using the program HKL2000,[S3] and further

processed using CCP4 package.[S4] The reported structure of the met form of WT (met-WT) (PDB code 3AGT) was used as a starting model for rigid-body refinement by REFMAC5.[S5] The model was refined with multiple rounds of manual rebuilding using Coot,[S6] and crystallographic refinement at a 1.9 Å resolution. The data collection and refinement statistics are listed in Table S1. Figs depicting the structure were prepared with PYMOL (http://www.pymol.org). The atomic coordinates and structure factors (PDB code 3WHN) have been deposited into the Protein Data Bank, http://www.rcsb.org/.

Computational Procedure

The broken-symmetry DFT calculations were performed on the models of I119H, in which H118 or H119 coordinates to Fe1, with Gaussian09 program package.[S7] In the H118 coordination model (H118on/H119off model), the atomic coordinates of the active site, which is composed of two iron atoms, the bridging E63 and D123, the five His residues coordinating to the iron atoms (H23, H59, H78, H82, H118), I119, and a chloride ion were extracted from the three-dimensional atomic structures of Cl^--bound met-WT determined at 1.4 Å resolution (PDB code: 3AGT),[S1] and I119 was replaced with His. The H119 coordination model (H118off/H119on model) was constructed using the three-dimensional atomic structures of the two iron atoms, E63, D123, H23, H59, H78, H82, H119, M120, and a chloride ion of the three-dimensional atomic structures of the Cl^--bound met-I119H mutant determined at 1.9 Å resolution (PDB code: 3WHN).[S1] In both models, the Cα atom is replaced with a CH_3 group.

The DFT calculations were performed with the B3LYP exchange-correlation functionals.[S8,S9] Tatewaki−Huzinaga MIDI (533(21)/53(21)/(41))[S10] plus Hay's *d* diffuse functions ($\alpha = 0.1215$)[S11] for the iron atoms and Pople's 6-31G(d) basis sets for other atoms[S12,S13] (BSI) were used for geometry optimization. Zero point energies and thermal corrections at 298 K (scaled by 0.98)[S14] were included in the reported energies. Wachters+f basis sets[S15] for the iron atoms and Pople's 6-311++G(df,pd) basis sets[S16] for other atoms (BSII) were utilized for single-point electronic structure calculations. The environmental effect inside the protein was computed with a polarized continuum model (PCM)[S17] using UAKS cavity[S18] with a dielectric constant of 4.0.[S19] The Cα and Cβ atoms of the coordinating residues were fixed during geometry optimization to preserve the structure and emulate the partially constraining effect of the protein environment.

The interaction energies (E_{int}) were evaluated between the first coordination sphere, which is composed of the two iron atoms, the chloride ion, the bridging residues, and five His residues coordinating to the iron atoms, and His119 for the H118on/H119off model or Met120 for the H118off/H119on model. We used the following equation to compute the interaction energies in the gas phase at the B3LYP/BSII level of theory:

$$E_{int} = E_{model} - (E_{1st} + E_X) ,$$

where E_{model} denotes the total energy of the H118on/H119off model or the H118off/H119on model, E_{1st} represents the total energy of the first coordination sphere, and E_X is the total energy of His119 in the H118on/H119off model or Met120 in the H118off/H119on model. The geometries of the first coordination sphere, His119, and Met120 were extract from the optimized structures of the H118on/H119off and H118off/H119on models.

Resonance Raman Spectroscopy

Resonance Raman scattering was excited at 488.0 nm with an Ar^+ laser (Spectra Physics, 2017) and detected with a CCD (Princeton Instruments) attached to a triple polychrometer (JACSO, NR-1800). The slit width was set to 200 µm. The laser power was 100 mW at the sample point. The spectra were collected at 25 ºC with a spinning cell. The concentration of all samples was controlled to be 1 mM. Toluene and acetone were used as references. Accuracy of the peak positions of the Raman bands was ±1 cm^{-1}.

FTIR Spectroscopy

The azide adduct of met-WT and met-I119H were prepared by mixing protein solution (1 mM) in 50 mM HEPES (pH 7.0) with 0.9 eq of sodium azide at 4 ºC for 12 h. The formation of the azide adduct was confirmed by increase of the absorption at 443 nm for met-WT and 425 nm for met-I119H, respectively. A 10 µL droplet of the solution of the azide adduct was sandwiched between two CaF_2 windows with a 25 µm polypropylene spacer. The cryostat was installed in the FTIR sample compartment and kept in the temperature dropped to 5 K. A series of FTIR spectra of 512 scan accumulations were collected with a 4 cm^{-1} resolution using a FT/IR-6100 type A (JASCO) equipped with liquid N_2 cooled MCT detector.

This journal is © The Royal Society of Chemistry 2014

H_2O_2 Consumption

The amount of unreacted H_2O_2 was determined by titration with iodide ion after mixing 2 μL of 500 mM H_2O_2 with 200 μL of 100 μM protein solution in 50 mM HEPES (pH 7.0). Each 10 μL of the reaction mixture was added to 1 mL of 1 M NaI_{aq} after 1, 3, 5, 10, 15, 30, 60, 120, and 180 min. The amount of I_3^- was determined by the absorption (λ_{max} 350 nm, $\varepsilon = 3.1 \times 10^4$ M^{-1} cm^{-1}).

Evolution of O_2 gas was identified using a Shimadzu GC-2014 gas chromatograph with a TCD detector equipped with a Molecular Sieve 5A column. In the glove box, 10 μL of 500 mM H_2O_2 was added to 1 mL of 100 μM WT in 50 mM HEPES (pH 7.0). The head-space gas (100 μL) of the reaction mixture was sampled by a gastight syringe after 3 h in the glove box. The gas was applied to GC equipped with an activated charcoal column and the TCD detector to determine the amount of generated O_2, which was detected with the retention time of 1.40 min.

Met-WT (50 μM) was anaerobically reduced to the deoxy form by the addition of 20 eq of sodium dithionite and 5 eq of methylviologen at 25 ºC. The solution was incubated for 3 h in the glovebox under an N_2 atmosphere. The resulting solution was passed over a HiTrap desalting column (GE Healthcare) equilibrated with degassed 50 mM HEPES (pH 7.0), and the prepared the deoxy form was mixed with 1.2 eq or 10 eq H_2O_2 under the N_2 atmosphere.

Oxidation Reaction of Guaiacol and 1,4-Cyclohexadiene

The oxidation reaction of guaiacol by WT and I119H was followed by time-course measurement of UV−vis spectra at 25 ºC. A mixture of the protein and guaiacol in MeOH was rapidly mixed with H_2O_2 in HEPES buffer (pH 7.0). The final concentrations are as follows: [Protein] = 100 μM, [2-guaiacol] = 5 mM, and [H_2O_2] = 5 mM. The oxidation reactions were monitored by observing the increase absorbance at 470 nm (λ_{max} 470 nm, $\varepsilon = 2.6 \times 10^4$ M^{-1} cm^{-1}).[S20]

The oxidation of 1,4-cyclohexadiene (CHD) was carried out in 50 mM HEPES (pH 7.0) at 25 ºC. A buffer solution of the protein, CHD, and ethylbenzene (internal standard) was incubated prior to the addition of H_2O_2 to initiate the reaction. [Protein] = 100 μM, [CHD] = 5 mM, [ethylbenzene] = 0.5 mM, and [H_2O_2] = 5 mM. After the reaction period of 3 h, cooled Et_2O and saturated $NaCl_{aq}$ was added, and the reaction mixture was vigorously shaken using a vortex mixer to extract the organic materials. The separated organic phase was analyzed with a GCMS-QP2010 Ultra equipped with a DB-1 column.

Table S1 Statistics of X-ray crystallographic data and structure refinement

Data collection	
Beamline	SPring-8 BL41XU
Wavelength (Å)	1.0
Resolution (Å)[a]	30−1.9 (1.97−1.90)
Space group	*P*1
Cell dimensions	
a, *b*, *c* (Å)	33.57, 44.08, 46.72
α, β, γ (°)	87.3, 77.4, 90.0
Total reflections	79685
Unique reflections	20052
R_{sym} (%) [a,b]	9.8 (32.3)
Completeness (%) [a]	97.9 (96.4)
$I/\sigma(I)$ [a]	14.3 (4.2)
Redundancy [a]	4.0 (4.0)
Refinement	
Resolution range (Å)	20−1.9
R/R_{free} factor (%)	19.3/24.7
R.m.s. deviation	
bond lengths (Å)	0.012
bond angles (˚)	1.36
Ramachandran (%)	
favored	97.7
allowed	2.3
outlier	0

[a]Numbers in parentheses are for the highest-resolution shell.

$R_{sym} = \Sigma_{hkl}\Sigma_i|I_i(hkl) - \langle I(hkl)\rangle| / \Sigma_{hkl}\Sigma_i I_i(hkl)$ where $\langle I(hkl)\rangle$ is the average intensity of the *i* observations. $R = \Sigma_{hkl}|F_{obs}(hkl) - F_{calc}(hkl)|/\Sigma_{hkl}F_{obs}(hkl)$, where F_{obs} and F_{calc} are observed and calculated structure factors, respectively. R_{free} was calculated with 5% of the reflections.

Table S2 Selected bond distances and angles

	met-I119H	met-WT[a]	met-Hr[a]	met-Hr(OH)[a]
Fe1−Fe2 (Å)	3.2	3.3	3.2	3.3
Fe1−μO (Å)	1.9	1.9	1.8	1.8
Fe2−μO (Å)	1.9	2.0	1.8	1.8
Fe2−X[b] (Å)	2.4	2.4	2.4	2.2
Fe1−Nε(H78) (Å)	2.4	2.3	2.2	2.2
Fe1−Nε(H82) (Å)	2.3	2.2	2.2	2.2
Fe1−Nε(H118 or H119)[c] (Å)	2.2	2.2	2.2	2.2
Fe2−Nε(H23) (Å)	2.4	2.2	2.2	2.2
Fe2−Nε(H59) (Å)	2.2	2.2	2.2	2.2
Fe1−Oε1(E63) (Å)	2.2	2.2	2.1	2.1
Fe2−Oε2(E63) (Å)	2.6	2.5	2.2	2.1
Fe1−Oδ1(D123) (Å)	2.3	2.1	2.1	2.1
Fe2−Oδ2(D123) (Å)	2.1	2.1	2.1	2.1
Fe1−μO−Fe2 (deg)	115	115	128	126

[a] The distances and the angle of met-WT, met-Hr, and met-Hr(OH) were obtained from the reported structures in PDB entries 3AGT, 1A7D, and 1A7E, respectively. The values of met-I119H are from this work.

[b] X is Cl for met-WT and met-Hr, and OH for met-Hr(OH).

[c] H118 for met-WT and H119 for met-I119H.

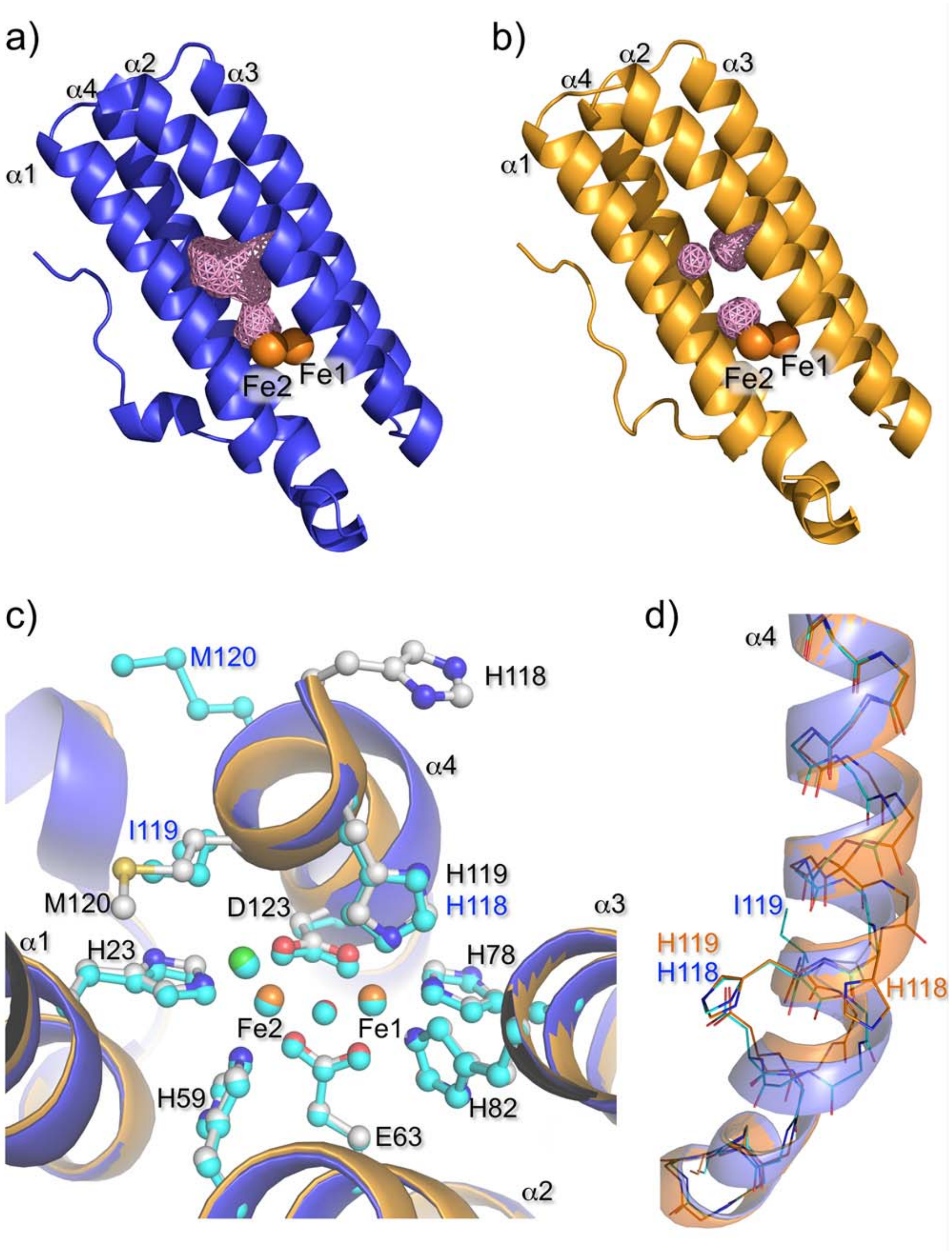

Fig. S1 Overall structures of (a) met-WT and (b) met-I119H. The pink grids represent the hydrophobic cavities. (c) Superimposition of the diiron site structures of met-I119H (carbon in white, oxygen in red, nitrogen in blue, and chloride in green) and met-WT (blue). (d) Superimposition of the α4 helix of met-I119H (orange) and met-WT (blue).

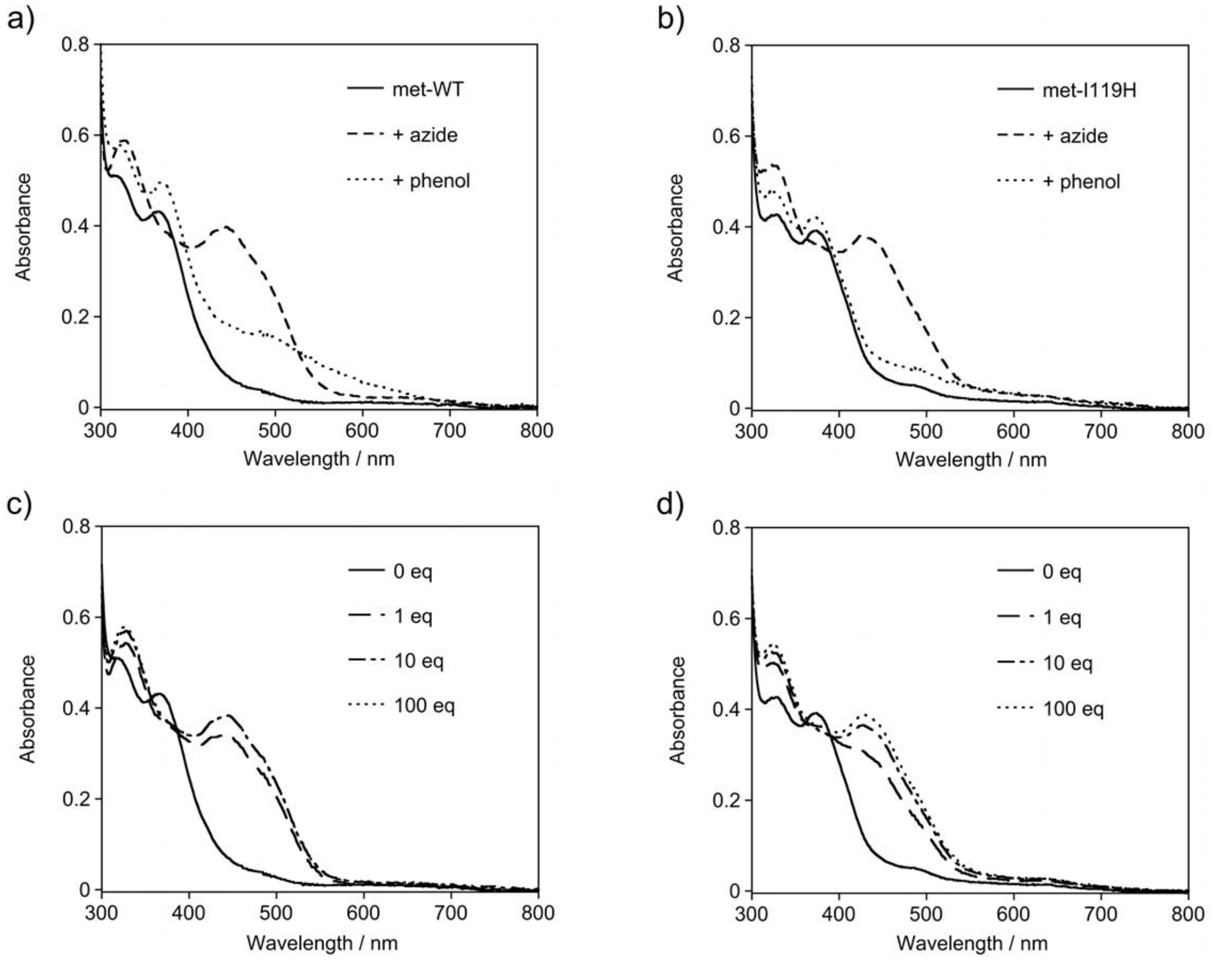

Fig. S2 UV−vis absorption spectra of (a) met-WT and (b) met-I119H, and their adducts with exogenous ligands in 50 mM HEPES (pH 7.0). The spectra of proteins (100 μM) in the absence of ligands (solid lines), in the presence of 5 mM NaN_3 (dashed lines), and 5 mM phenol (dotted line) are shown. UV−vis absorption spectra of the azide adduct of (c) met-WT and (d) met-I119H in 50 mM HEPES (pH 7.0) in the presence of different equivalent of sodium azide (1, 10, and 100 eq). The proteins (100 μM) were incubated for 12 h at 4 ºC before the measurements.

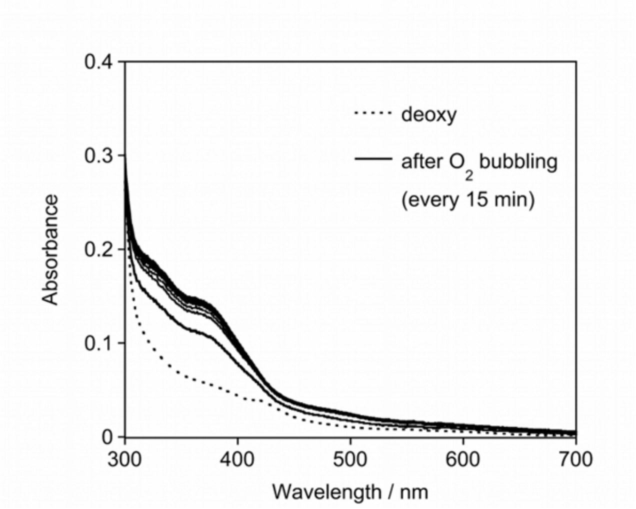

Fig. S3 (a) Time course absorption changes of deoxy form of I119H (50 μM) reacted with O_2 by air-bubbling in 50 mM HEPES (pH 7.0) at 25 ºC. The spectra was recorded every 15 min and the spectrum of deoxy form was shown as a dotted line.

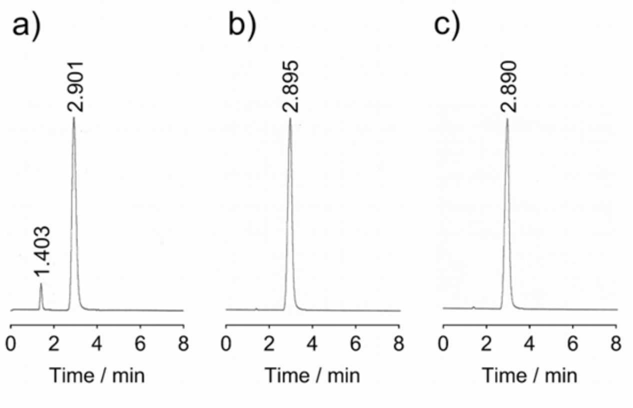

Fig. S4 Gas chromatogram profiles obtained by the reactions of H_2O_2 with met-WT. (a) met-WT in the presence of H_2O_2, (b) met-WT in the absence of H_2O_2, and (c) H_2O_2 without the protein. Reaction conditions: [met-WT] = 100 μM, [H_2O_2] = 5 mM; 50 mM HEPES (pH 7.0) at 25 ºC under N_2 atmosphere. The gases, O_2 and N_2, were detected at the retention time of 1.40 min and 2.90 min, respectively.

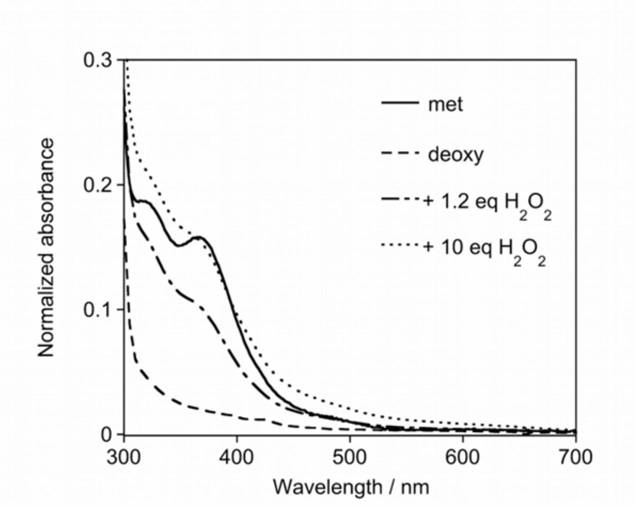

Fig. S5 UV−vis absorption spectra of met form (solid line) and deoxy form (dashed line) of WT. The spectra of deoxy form after the addition of 1.2 eq (dashed dotted line), and 10 eq (dotted line) of H_2O_2 are also shown. [protein] = 50 μM, in 50 mM HEPES (pH 7.0) at 25 ºC.

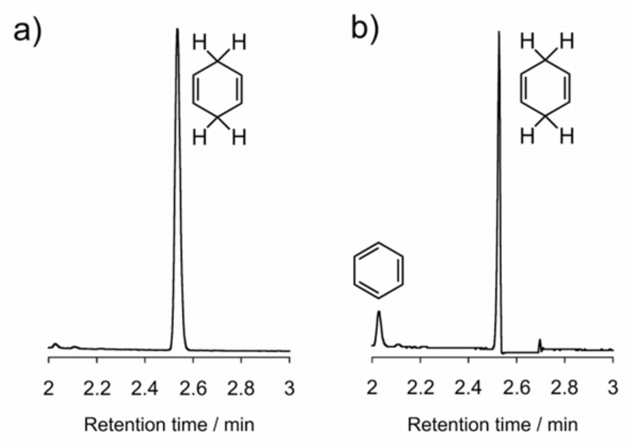

Fig. S6 GC-MS profiles of the oxidation product of 1,4-cyclohexadiene (CHD) using (a) met-WT and (b) met-I119H. Reaction conditions; [protein] = 100 μM, [CHD] = 5 mM, [H_2O_2] = 5 mM; 50 mM HEPES (pH 7.0) at 25 ºC.

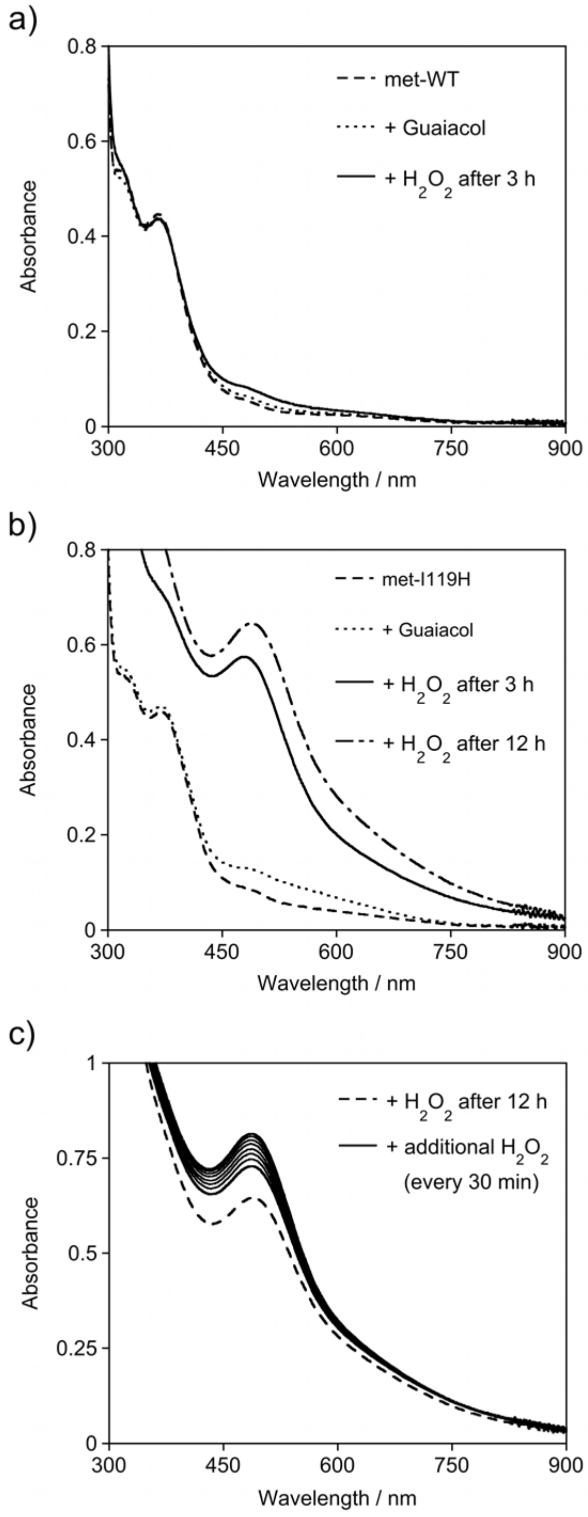

Fig. S7 UV−vis absorption spectra following the oxidation reaction of guaiacol by (a) met-WT, and (b) the met-I119H. The spectra before the reaction (dashed line), after the addition of guaiacol (dotted line), after 3 h (solid line), and after 12 h (dashed and dotted line). (c) The spectra of met-I119H 12 h after the first addition of H_2O_2 (dashed line) and after the second addition of H_2O_2 (solid line). [protein] = 100 μM, [2-guaiacol] = 5 mM, [H_2O_2] = 5 mM; 50 mM HEPES (pH 7.0) at 25 ºC.

This journal is © The Royal Society of Chemistry 2014

Scheme S1 Plausible reaction mechanism of the consumption of H_2O_2 by wild type DcrH-Hr

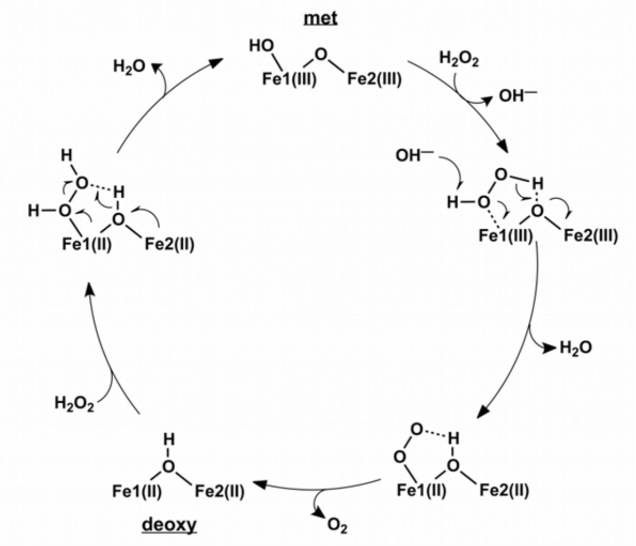

References

S1 A. Onoda, Y. Okamoto, H. Sugimoto, Y. Shiro and T. Hayashi, *Inorg. Chem.*, 2011, **50**, 4892.

S2 L. L. Stookey, *Anal. Chem.*, 1970, **42**, 779.

S3 Z. Otwinowski and W. Minor, *Methods Enzymol.*, 1997, **276**, 307. http://krzys.med.virginia.edu/CrystUVa/wladek_processing.php (accessed Jan 16, 2004).

S4 Collaborative Computational Project, Number 4 “The CCP4 suite, programs for protein crystallography” *Acta Crystallogr. D*, 1994, **50**, 760.

S5 G. N. Murshudov, A. A. Vagin and E. J. Dodson, *Acta Crystallogr. D*, 1997, **53**, 240.

S6 P. Emsley and K. Cowtan, *Acta Crystallogr. D*, 2004, **60**, 2126.

S7 Gaussian 09, Revision C.1, M. J. Frisch, G. W. Trucks, H. B. Schlegel, G. E. Scuseria, M. A. Robb, J. R. Cheeseman, G. Scalmani, V. Barone, B. Mennucci, G. A. Petersson, H. Nakatsuji, M. Caricato, X. Li, H. P. Hratchian, A. F. Izmaylov, J. Bloino, G. Zheng, J. L. Sonnenberg, M. Hada, M. Ehara, K. Toyota, R. Fukuda, J. Hasegawa, M. Ishida, T. Nakajima, Y. Honda, O. Kitao, H. Nakai, T. Vreven, J. A. Montgomery, Jr., J. E. Peralta, F. Ogliaro, M. Bearpark, J. J. Heyd, E. Brothers, K. N. Kudin, V. N. Staroverov, R. Kobayashi, J. Normand, K. Raghavachari, A. Rendell, J. C. Burant, S. S. Iyengar, J. Tomasi, M. Cossi, N. Rega, J. M. Millam, M. Klene, J. E. Knox, J. B. Cross, V. Bakken, C. Adamo, J. Jaramillo, R. Gomperts, R. E. Stratmann, O. Yazyev, A. J. Austin, R. Cammi, C. Pomelli, J. W. Ochterski, R. L. Martin, K. Morokuma, V. G. Zakrzewski, G. A. Voth, P. Salvador, J. J. Dannenberg, S. Dapprich, A. D. Daniels, Ö. Farkas, J. B. Foresman, J. V. Ortiz, J. Cioslowski and D. J. Fox, Gaussian, Inc., Wallingford CT, 2009.

S8 A. D. Becke, *J. Chem. Phys.*, 1993, **98**, 5648.

S9 C. Lee, W. Yang and R. G. Parr, *Phys. Rev. B.* 1988, **37**, 785.

S10 H. Tatewaki and S. Huzinaga, *J. Chem. Phys.*, 1980, **72**, 399.

S11 P. J. Hay, *J. Chem. Phys.*, 1977, **66**, 4377.

S12 T. Clark, J. Chandrasekhar, G. W. Spitznagel and Shleyer, P. v. R. *J. Comp. Chem.*, 1983, **4**, 294.

S13 R. Ditchfield, W. J. Hehre and J. A. Pople, *J. Chem. Phys.*, 1971, **54**, 724.

S14 C. W. Bauschlicher and H. J. Partridge, *J. Chem. Phys.*, 1995, **103**, 1788.

S15 A. J. H. Wachters, *J. Chem. Phys*,. 1970, **52**, 1033.

S16 P. J. Hariharan and J. A. Pople, *Theor. Chem. Acta*, 1973, **28**, 213.

S17 M. Cossi, G. Scalmani, N. Rega and V. Barone, *J. Chem. Phys.*, 2002, **117**, 43.

S18 Y. Takano and K. N. Houk, *J. Chem. Theory Comput.*, 2005, **1**, 70.

This journal is © The Royal Society of Chemistry 2014

S19 M. K. Gilson and B. H. Honig, *Biopolymers*, 1986, **25**, 2097.

S20 G. D. DePillis, B. P. Sishta, A. G. Mauk and P. R. Ortiz de Montellano, *J. Biol. Chem.*, 1991, **266**, 19334.
